# VIRTUAL TRAINING FOR MANAGING SYMPTOMS OF DEMENTIA: A CULTURAL ADAPTATION FOR LATINO CAREGIVERS

**DOI:** 10.1093/geroni/igad104.2013

**Published:** 2023-12-21

**Authors:** Maggie Ramirez, Robert Penfold

**Affiliations:** University of Washington School of Public Health, Seattle, Washington, United States; Kaiser Permanente Washington, Seattle, Washington, United States

## Abstract

STAR-Caregivers Virtual Training and Follow-up (STAR-VTF) is an evidence-based intervention that teaches family caregivers how to manage behavioral and psychological symptoms of dementia. The study objective was to identify what adaptations to STAR-VTF are needed to improve cultural relevance for Latino caregivers. Thirty caregivers of people living with dementia who self-identify as Hispanic/Latino and 14 providers of healthcare and social services were interviewed. Interview transcripts were analyzed using thematic analysis methods. The Cultural Treatment Adaptation Framework guided data collection and analysis. Three themes were identified: (1) there was a need to increase awareness about dementia and decrease stigma; (2) semantics mattered as certain words and phrases could be stigmatizing, offensive, or culturally inappropriate; and (3) there was a need to incorporate into program materials the traditional family structure and nature of caregiving in Latino families. Adaptations were performed on STAR-VTF, including expanding content to improve understanding of dementia, revising language that was viewed as problematic, and adding cultural examples to reflect the range of family involvement in caring for people living with dementia and multigenerational living. Findings from this study advance understanding of the Latino caregiver experience and how to modify programs to better serve their needs.

